# Zika virus-like particles (VLPs) produced in insect cells

**DOI:** 10.3389/fphar.2023.1181566

**Published:** 2023-06-12

**Authors:** Renata Gois de Mello, Thaissa Consoni Bernardino, Luis Giovani Oliveira Guardalini, Renato Mancini Astray, Marta Maria Antoniazzi, Simone Gonçalves Silva Jared, Eutimio Gustavo Fernández Núñez, Soraia Attie Calil Jorge

**Affiliations:** ^1^ Laboratório de Biotecnologia Viral, Instituto Butantan, São Paulo, SP, Brazil; ^2^ Laboratório de Biologia Estrutural, Instituto Butantan, São Paulo, SP, Brazil; ^3^ Grupo de Engenharia de Bioprocessos, Escola de Artes, Ciências e Humanidades (EACH), Universidade de São Paulo, São Paulo, SP, Brazil

**Keywords:** virus-like particles, Zika virus, recombinant baculovirus, Sf9 cells, insect cells

## Abstract

**Introdutcion:** The Zika virus (ZIKV) infections are a healthcare concern mostly in the Americas, Africa, and Asia but have increased its endemicity area beyond these geographical regions. Due to the advances in infections by Zika virus, it is imperative to develop diagnostic and preventive tools against this viral agent. Virus-like particles (VLPs) appear as a suitable approach for use as antiviral vaccines.

**Methods:** In this work, a methodology was established to produce virus-like particles containing the structural proteins, C, prM, and E of Zika virus produced in insect cells using the gene expression system derived from baculovirus. The vector pFast- CprME -ZIKV was constructed containing the gene sequences of Zika virus structural proteins and it was used to generate the recombinant bacmids (Bac- CprME -ZIKV) through transformation into DH10Bac^TM^ cells. The Bac- CprME -ZIKV was transfected in Spodoptera frugiperda (Sf9) insect cells and batches of BV- CprME -ZIKV were obtained by infection assays using a multiplicity of infection of 2. The Sf9 cells were infected, and the supernatant was collected 96 h post-infection. The expression of the CprME -ZIKV protein on the cell surface could be observed by immunochemical assays. To concentrate and purify virus-like particles, the sucrose and iodixanol gradients were evaluated, and the correct CprME -ZIKV proteins’ conformation was evaluated by the Western blot assay. The virus-like particles were also analyzed and characterized by transmission electron microscopy.

**Results and discussion:** Spherical structures like the native Zika virus from 50 to 65 nm containing the CprME -ZIKV proteins on their surface were observed in micrographs. The results obtained can be useful in the development path for a vaccine candidate against Zika virus.

## Introduction

Arboviruses (arthropod-borne viruses) have become a public health problem in the tropical and subtropical regions of the world. The Zika virus (ZIKV) together with the Dengue (DENV) and Chikungunya (CHIKV) viruses are the most prevalent arboviruses in the Americas, and are responsible for health problems with economic and social consequences for populations living in endemic areas (Rodríguez-Morales et al., 2015). The ZIKV is transmitted to humans through the bite of mosquitos of genus *Aedes* (*Aedes aegypti* and *A. albopictus*). It was first isolated from primates, rhesus monkeys, in the Zika forest in Uganda, Africa, in 1947(Dick et al., 1952; Macnamara, 1954). The ZIKV is currently considered a public health problem because of its rapid geographical spread that has occurred first in the Asia-Pacific region and then in the Western Hemisphere in the past 14 years (Lanciotti et al., 2007; Heang et al., 2012; Buathong et al., 2015; Marcondes and Ximenes, 2015; Mccarthy, 2016; Sikka et al., 2016). In 2015, the ZIKV was first identified on the American continent, specifically in the northeast region of Brazil (Zanluca et al., 2015). That same year, a 20-fold increase in the incidence of cases of congenital malformation compared to previous years was reported concurrently with the ZIKV outbreak in northeastern Brazil (Victora et al., 2016). This led the World Health Organization (WHO) to declare the spread of ZIKV and its association with birth defects as a Public Health Emergency of International Concern (PHEIC) on 01 February 2016 (WHO, 2020).

Infection caused by the ZIKV presents a variety of symptoms, such as fever, headache, myalgia, conjunctivitis, skin rashes, and arthralgia. In addition to these symptoms, the ZIKV is also associated with neurological complications such as Guillain-Barré syndrome, meningitis, myelitis, and meningoencephalitis in adults (Musso et al., 2014; ECDC, 2020). This virus infection can lead to abortion in pregnant women and cause congenital malformation (microcephaly), brain calcification, and muscle degeneration in newborns (Paoh, 2016). There is no specific treatment for the ZIKV infection, and there are no antiviral drugs or vaccines available to date.

The advance of infections caused by the ZIKV, cases of microcephaly in newborns, and lack of specificity and/or delay in diagnosis have led to the need to develop tools to detect and combat the infection, such as a safe and effective vaccine. Vaccination is considered the most efficient method to prevent viral infections; however, the development of vaccines against emerging viral infections, as in the case of the ZIKV, can face several challenges with regard to safety, speed, and efficiency. Virus-like Particles (VLPs) have been studied as vaccine candidates for decades, and several vaccines have already been approved for commercialization, in addition to those that are in different stages of clinical studies (Mohsen et al., 2017). VLPs are formed from viral structural proteins that have the ability to self-assemble, forming structures similar to that of the parental virus. The vaccination strategy using VLPs emerges as an excellent platform to develop efficient vaccines, since they have the ability to induce strong immune response, allow the test of multiple antigens on their surface, and are associated with a safer production process (Liu et al., 2016).

Currently, various platforms for the production of VLPs are widely used, such as bacteria, yeasts, transgenic plants, and mammalian and insect cells (Fuenmayor et al., 2017). The baculovirus expression system produced in insect cells has shown to be a promising system because of its ability to provide post-translational modifications such as protein glycosylation and generate more complex VLPs when compared with other systems, in addition to presenting the ability to express high levels of heterologous proteins (Roldão et al., 2010; Rodríguez-Limas et al., 2013).

In this context, this study aimed to produce and characterize a particle similar to the ZIKV and express its structural proteins using a gene expression system derived from baculoviruses produced in insect cell cultures. We were able to assemble and express the ZIKV proteins to form VLPs that were similar to the ZIKV virions. The results presented herein show the initial development of a VLP particle that, with additional analysis, could be used as a future vaccine candidate.

## Materials and methods

### Cell culture and viruses

*Spodoptera frugiperda* (Sf9) cells were chosen for the experiments and cultured in Sf-900™ III insect medium (Gibco, NY, United States) at 28°C. The ZIKV strain IEC was obtained from *Institute Evandro Chagas* (GenBank accession no. KU365780.1) and used for the genomic RNA extraction. The inactivated ZIKV control (IN-ZIKV) was prepared using the IEC strain and amplified in Vero cells (ATCC-CCL-81, VA, United States) at a multiplicity of infection (MOI) of 0.1 and harvested 96 h post infection. After purification, the virus was inactivated with β-propiolactone and incubated in liquid nitrogen until use.

### Construction of recombinant baculovirus

The pFast- CprME -ZIKV vector was constructed from a commercial vector, pFastBac™ (Invitrogen, CA, United States), with the genes of the structural proteins of the ZIKV, a sequence under the control of the baculovirus polyhedrin promoter. The CprME -ZIKV sequence was obtained by extracting genetic material from the ZIKV strain IEC using a QIAmp Viral RNA kit (Qiagen, MD, United States) followed by real-time polymerase chain reaction (RT-PCR) of the material to amplify the genes of interest. This sequence was cloned into the pFastBac™ vector using an In-Fusion^®^ kit (Clontech, CA, United States). Once the pFast- CprME -ZIKV vector was obtained, it was transformed into the *Escherichia coli* DH10Bac™ strain of the commercial Bac-to-Bac system (Invitrogen, CA, United States), according to the manufacturer’s instructions, to generate the bacmid BAC- CprME -ZIKV. The recombinant bacmid was purified using a Maxiprep kit (Qiagen, MD, United States) according to the manufacturer’s instructions. BAC- CprME -ZIKV was transfected into Sf9 cells to generate the corresponding recombinant baculovirus BV- CprME -ZIKV.

### Immunofluorescence and Western blot analyses of E-ZIKV protein expression

To detect the expression of the CprME -ZIKV protein, Sf9 cells were infected with the recombinant baculovirus BV- CprME -ZIKV using 1 × 10^6^ cells/well in a 6-well assay plate. Infection was performed using an MOI of 2, based on results obtained from previous tests, and the supernatant was harvested 72 h post infection. For the immunofluorescence assay, 5 × 10^4^ cell lysates of the infection were fixed on slides with methanol-acetone (1:1) at 4°C for 10 min. The fixed cells were washed with phosphate buffered saline (PBS) (0.1 M NaCl, 0.002 M KCl, 0.01 M NaHPO, and 0.001 M KH_2_PO_4_) and incubated with anti-E 4G2 (Mab 4G2) monoclonal antibody (1:100) at 4°C for 90 min. After that, the cells were washed again and incubated with anti-mouse IgG-fluorescein isothiocyanate secondary antibody (1:500) (FITIC; Abcam, United Kingdom) at 4°C for 1 h. The samples were observed and digitally photographed under a fluorescence microscope (BX51, Olympus, JP). For the Western blot analysis, the samples were lysed with radioimmunoprecipitation assay (RIPA) buffer, loaded on a 12% SDS-PAGE gel by electrophoresis, and the gels were electroblotted onto a 0.45 µm nitrocellulose membrane (GE Healthcare, IL, United States) using a Mini Trans Blot Cell system (BIO-RAD, CA, United States). The membranes were blocked in 3% (W/V) skimmed milk for 1 h, washed with PBS three times for 10 min each, and incubated with Mab 4G2 (1:100) for 90 min. After that, the membranes were washed again and incubated with HRP-conjugated goat anti-mouse secondary antibody (1:2000) for 1 h. Protein band signals were detected using SuperSignal West Pico Chemiluminescent Substrate (Thermo Scientific, IL, United States) and photo-documented using the UVITEC Cambridge software with analysis in the Alliance application (Uvitec Cambridge, CB, United Kingdom).

### Production and purification of ZIKV-VLPs

For production of the VLPs, infection assays were performed on Sf9 cell line in suspension using 1 × 10^6^ cells/mL with a total volume of 20 mL in 100 mL shaken flasks of cell culture. The cells were infected with the recombinant baculovirus BV- CprME -ZIKV with a MOI of 2 and the flasks were incubated in an orbital shaker at 28°C at 100 rpm shaking. The cells were harvested at 96 h post infection (h.p.i.) based on the results obtained from previous tests. The cell culture supernatant was clarified by centrifugation at 1,300 g for 15 min, recovered, and filtered through a 0.45 µm mesh. The clarified supernatant was ultracentrifuged at 1,50,000 g at 4°C for 90 min (MLA-50 rotor; Beckman Coulter, United States) and the generated pellet was resuspended in PBS and kept overnight (16 h). After the concentration step, the pellet was layered onto a 20% sucrose cushion and ultracentrifuged at 1,50,000 g for 3 h (MLS-50 rotor; Beckman Coulter). The supernatant was discarded and the pellet was resuspended in PBS and kept overnight (16 h). For the purification step, two gradient procedures were chosen: the sucrose gradient, in which the pellets were subjected to a continuous sucrose gradient (10%–60%) and ultracentrifuged at 1,50,000 g for 3 h (MLS-50 rotor; Beckman Coulter), and the iodixanol gradient, in which the pellets were subjected to a continuous iodixanol gradient (10%–40%) and ultracentrifuged at 1,50,000 g for 3 h. Fractions of 500 µL from both gradients were collected from the tubes for further analysis.

### Dot blot and Western blot analyses of CprME -ZIKV protein expression in ZIKV-VLPs

For the Dot blot assay, fractions generated from both the sucrose and iodixanol gradients were subjected to lysis using RIPA buffer (150 mM NaCl, 1% IGEPAL, 0.5% sodium deoxycholate, 50 mM Tris, pH 8.0) with a ratio of 1:10 (buffer: sample) and incubated at 4°C for 30 min. After this process, 50 µL of the samples were applied onto a 0.45 µm nitrocellulose membrane (GE Healthcare, NJ, United States). The membrane was then incubated in a 3% (W/V) skimmed milk blocking solution for 1 h, washed three times with PBS for 10 min, and incubated under agitation with the Mab 4G2 (1:100) or the anti-gp64-baculovirus (Mab gp64) monoclonal antibody (1:2000) for 90 min. The membranes were then washed three times each with PBS for 10 min and incubated under agitation with HRP-conjugated goat anti-mouse secondary antibody (1:2000) for 1 h. The membrane was developed with the SuperSignal West Pico Chemiluminescent Substrate (Thermo Scientific, IL, United States) on the photo-documenter Uvitec Cambridge, with analysis in the Alliance program (Uvitec Cambridge, CB, United Kingdom) For the Western blot assay, the same methodology described for detection of CprME -ZIKV protein expression in infected Sf9 cells was followed.

### Characterization of VLPs by transmission electron microscopy

Samples collected from the sucrose and iodixanol gradients were analyzed by transmission electron microscopy (TEM) to evidence the presence of VLPs. For the negative staining assay, 10 µL of the samples were dripped onto carbon-coated copper grids (300 mesh), allowed to adsorb for 1 min, and the excess was removed with filter paper. After that, 10 µL of 2% uranyl acetate solution was applied over the sample for 1 min and the excess was removed. The negatively stained sample was analyzed and documented under a Zeiss Leo 906 E electron microscope (Germany). For immunoelectron microscopy (IEM), 10 µL of the samples were loaded onto carbon-coated nickel grids (400 mesh), incubated at room temperature for 10 min, blocked with 1% BSA for 5 min, and incubated with MAb 4G2 at room temperature for 2 h. The grids were then washed three times with PBS and incubated with the goat anti-mouse IgG secondary antibody, Alexa Fluor 488–10 nm colloidal gold (Invitrogen, CA, United States) for 1 h. The grids were washed two times with PBS, negatively stained with 2% uranyl acetate solution, and examined by TEM as previously described. To analyze the production of VLPs within the infected Sf9 cells, the ultrastructural cytochemical technic was performed. For that, cells infected with BV- CprME -ZIKV were harvest 72 h.p.i. and were fixed with 2.5% glutaraldehyde, 2% paraformaldehyde in 0.1 M cacodylate buffer, pH 7.2 and incubated at 4°C for 24 h. The pellets were washed 3x with 0.1 M sodium cacodylate for 15 min each and post-fixed with 1% osmium tetroxide in 0.1 M cacodylate buffer for 1 h at room temperature. The pellets were washed 3x with 1 M sodium cacodylate for 15 min each. Cells were dehydrated in a graded series of ethanol following the cycle: 2% × 70% ethyl alcohol/10 min; 2% × 95% ethyl alcohol/15 min; 3x absolute ethyl alcohol/20 min; 1x propylene oxide + absolute ethyl alcohol (1: 1)/15 min; 2x propylene oxide/15 min; 1x propylene oxide + resin/2 h. The sample was pre-embedded in pure resin Embed 812 (EMS, Hatfield, PA) and incubated overnight at room temperature. All steps were done under agitation. The material was processed in the vacuum chamber and was incubated at 60°C for 48 h. Ultrathin cuts were made from the blocks and they were layered in 300 mesh copper metallic grids pre-coated with 2% Parlodion and carbon. Ultrathin sections were stained with 2% uranyl acetate and alkaline lead citrate. Grids were examined with TEM.

## Results

### Expression of CprME -ZIKV proteins on Sf9 cells infected with BV- CprME -ZIKV

**FIGURE 1:**
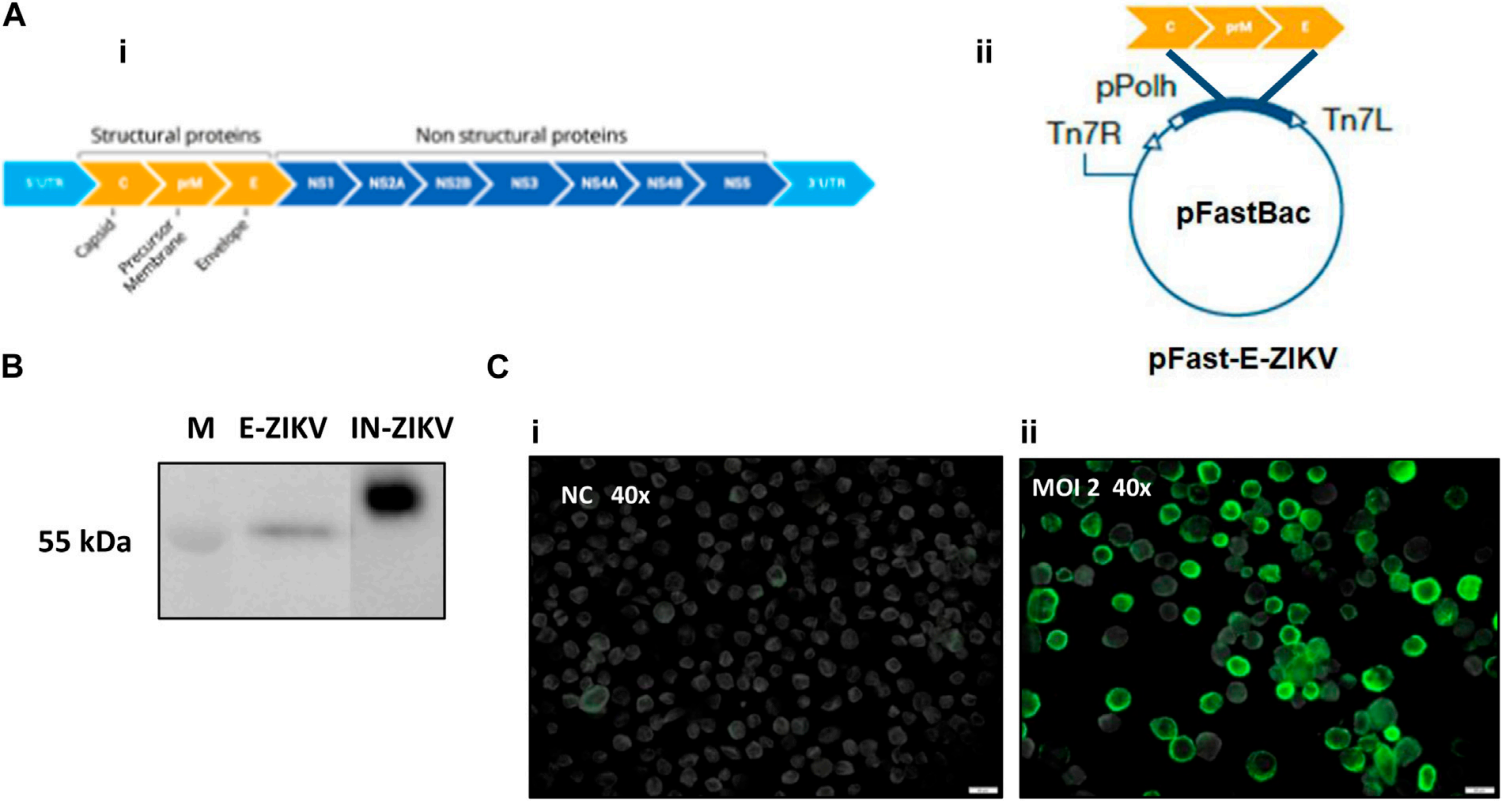
Construction of recombinant baculoviras BV-E-ZIKV and analysis of protein E-ZIKV expression. **(A)** (i) Schematic representation of the ZEKV polyprotein; **(A)** (ii) Structure of the vector pFast-E-ZIKV encoding C-prM-E under the control of a polyhedrin promoter (pPolh) for the generation of BV-E-ZIKV; **(B)** Western blot analysis of the E-ZIKV protein produced by Sf9 cells infected with BV-E-ZIKV, Inactivated ZIKV (IN-ZIKV) was used as the positive control; **(C)** (i) Sf9 cells not infected were used as negative control (NC); **(C)** (ii) Immunofluorescence assay showing E-ZIKV proteins in Sf9 cells surface infected with MOI of 2 of the BV-E-ZIKV. Scale bars = 20 μm.

To generate the recombinant BAC- CprME -ZIKV bacmid, the RNA fragment encoding CprME -ZIKV proteins was inserted into the pFastBac™ vector under the control of the polyhedrin promoter (Figure 1A). The recombinant BV- CprME -ZIKV baculovirus was generated after transfection of BAC- CprME -ZIKV on Sf9 cells. The CprME -ZIKV protein expression was confirmed by Western blot and immunofluorescence analyses. Western blot analysis revealed expression of a 55 kDa size band that corresponded with the predicted size of the E protein of parental ZIKV as shown in the positive control (IN-ZIKV) (Figure 1B). Through the immunofluorescence procedure, it was possible to observe the CprME -ZIKV protein expression on the surface of Sf9 cells infected with BV- CprME -ZIKV at a MOI of 2 and collected at 72 h.p.i., (Figure 1C).

### Expression of CprME -ZIKV protein on ZIKV-VLPs

**FIGURE 2:**
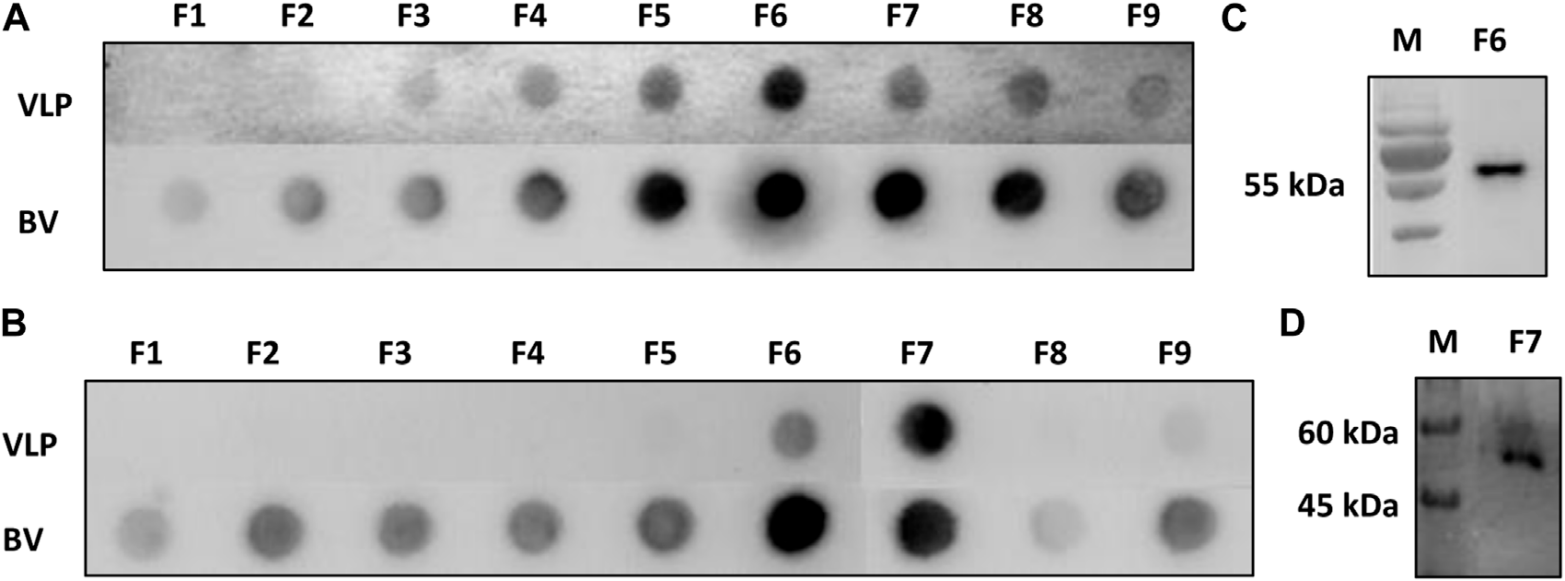
Analysis of protein E-ZIKV expression on purified VLPs. **(A)** Dot blot of the fractions collected from the sucrose gradient (Fl-F9) marked with monoclonal antibody 4G2 (VLP-ZIKV) and monoclonal antibody gp64 (BV-E-ZIKV); **(B)** Dot blot of the fractions collected from the iodixanol gradient (Fl-F9) marked with monoclonal antibody 4G2 (VLP-ZIKV) and monoclonal antibody gp64 (BV-E-ZIKV); **(C)** Western blot analysis of purified fraction F6 collected from the sucrose gradient showing protein E of the expected size of −55 kDa, M: Marker TrueColor Protein Marker (Synapse); **(D)** Western blot analysis of purified fraction F7 collected from iodixanol gradient fraction showing protein E of the expected size of −55 kDa. For the Western blot analysis, the primary monoclonal antibody anti-E 4G2 was used. M: Marker: PageRuler^TM^ Plus (Thermo Fisher Scientific).

Sf9 cell cultures infected with BV-E-ZIKV were collected at 96 h.p.i., clarified, filtered, and then subjected to a process of concentration and purification of the VLPs, as described in the Material and Methods section. After the purification procedure, nine fractions of 500 µL of the generated gradient were collected and subjected to analysis of purity and identity of the CprME -ZIKV protein composing the purified VLPs by Dot blot and Western blot analyses. The Dot blot analysis was performed using the 4G2 anti-E-flavivirus monoclonal antibody to stain the produced VLPs and the anti-gp64-baculovirus monoclonal antibody to stain the produced baculovirus. In fractions of the sucrose gradient marked with MAb 4G2, it was possible to observe a more intense staining in fraction F6 (Figure 2A-VLP), where it was also possible to observe a halo in the gradient, thus evidencing a higher concentration of the CprME -ZIKV protein in that fraction. In fractions marked with MAb gp64, it was possible to observe a more intense staining in the F6 and F7 fractions, evidencing a higher concentration of baculoviruses in the same fractions where the VLPs were also more concentrated (Figure 2A-BV). In fractions of the iodixanol gradient marked with MAb 4G2, it was possible to observe a more intense staining in fraction F7 (Figure 2B-VLP), where it was possible also possible to observe a halo in the gradient, evidencing a higher concentration of the CprME -ZIKV protein in that fraction (Figure 2B-BV). In fractions marked with MAb gp64, it was possible to observe a more intense staining in the fraction F6, evidencing a higher concentration of baculoviruses in that fraction. The Western blot analysis was performed with fraction F6 of the sucrose gradient and fraction F7 of the iodixanol gradient. The Western blot analysis revealed the CprME -ZIKV protein at the expected size of ∼55 kDa (Figures 2C, D), which correlated with the E protein size of the parental ZIKV in samples of both gradients.

### Characterization of ZIKV-VLPs

**FIGURE 3:**
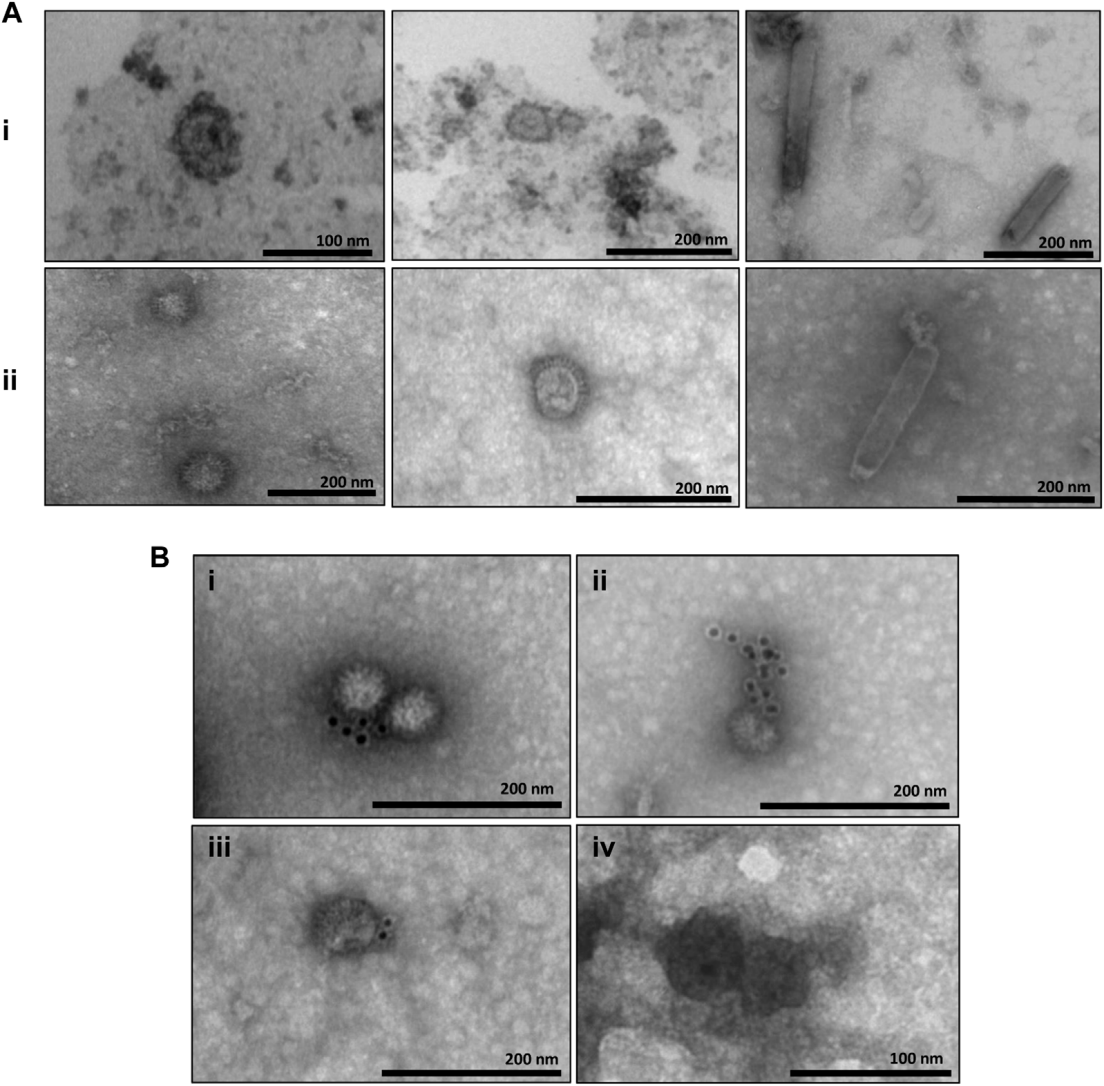
Characterization of VLPs by transmission electron microscopy and IEM. **(A)** (i) Negative staining of the E enriched fraction from the sucrose gradient showing structures similar to VLPs-ZIKV (50–65 nm) and baculovirus structures (150–250 nm); **(A)** (ii) Negative staining of the E enriched fraction from the iodixanol gradient showing structures similar to VLPs-ZIKV (50–65 ma) and baculovitus structures (−300 nm); **(B)** EM of samples purified by the iodixanol gradient (i–iii) and the sucrose gradient (iv) showing structures similar to VLPs-ZIKV immunostained with gold particles. Scale bars are shown for each electron micrograph.

**FIGURE 4:**
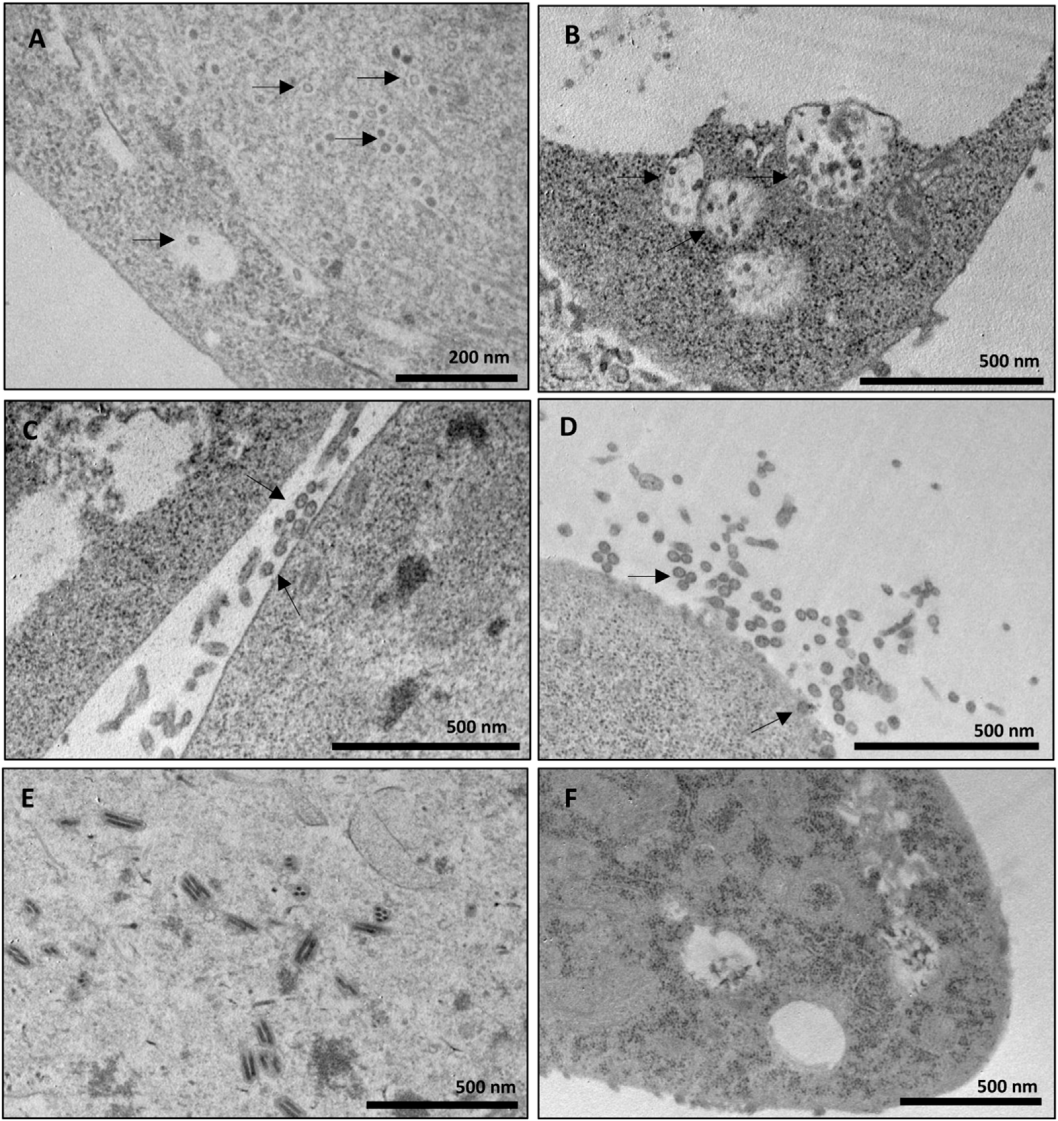
Ultrastructural cytochemical characterization of VLPs on Sf9 cells. **(A–E)** Transmission electron micrographs of Sf9 cells infected with BV-E-ZIKV. **(A)** Spherical particles presumed to be VLPs-ZIKV in the cell cytoplasm (black arrows); **(B)** Intracellular vesicles filled with small spherical particles (black arrows) in Sf9 cells; **(C,D)** Spherical particles presumed to be VLPs-ZIKV out of the cell membrane after budding (black arrows); **(E)** Baculovirus occlusion bodies being produced in the cell nucleus; **(F)** Sf9 cells not infected used as negative control presenting empty intracellular vesicles. Scale bars are shown for each electron micrograph.

The samples corresponding to the fractions collected in the sucrose and iodixanol gradients that showed more intense staining in the Dot blot analysis and in which the size of the CprME -ZIKV protein was confirmed by the Western blot analysis were subjected to analysis of generation of ZIKV-VLPs by TEM. To this end, the techniques of negative staining, IEM, and ultrastructural cytokine were used to characterize the VLPs. The negative staining assay revealed homogeneous spherical particles with diameters ranging from 50 to 65 nm in both the sucrose (Figure 3Ai) and iodixanol gradients (Figure 3Aii). It was also possible to observe baculovirus structures in the analyzed fractions of both gradients. Through IEM, it was possible to observe structures similar to VLPs in both gradients immunostained with the gold particles of the secondary antibody used in the technique, evidencing the presence of the E-ZIKV protein on the surface of the ZIKV-VLPs (Figure 3B). For the ultrastructural cytochemical characterization of VLPs within the Sf9 cells infected with the BV- CprME -ZIKV, the cells were harvested at 72 h.p.i. and analyzed using TEM. It was possible to observe spherical structures similar to VLPs in the cell cytoplasm (Figure 4A) and in vesicles of the Sf9 infected cells ranging from 30 to 40 nm in diameter (Figure 4B). Some particles ranging from 50 to 60 nm in diameter (Figures 4C, D) were also found in the cell outer membrane. It was also possible to observe some baculovirus occlusion bodies being produced in the nuclei of the Sf9 cells infected with BV- CprME -ZIKV (Figure 4E). The Sf9 cells used as control showed empty vesicles, in contrast to what was observed in the Sf9 infected cells (Figure 4F).

## Discussion

Until the last epidemiological update issued by the WHO in July 2019, 87 countries and territories had reported autochthonous transmission of the ZIKV. The ZIKV is transmitted by *A. aegypti* mosquitoes, and there is still no efficient control of their infestation in the regions where they are considered an established vector. The recent outbreaks of other arboviruses also transmitted by the same mosquito demonstrate that new ZIKV outbreaks may occur. In addition, infection caused by the ZIKV continues to carry the risk of complications such as adverse pregnancy outcomes with presence of congenital malformation and Guillain-Barré syndrome. Therefore, the development of tools that allow the proper combat of the ZIKV is extremely important. Although the development of a vaccine against the ZIKV disease using different platforms has been studied and cited in different studies, there are no vaccines commercially available to date (Lin et al., 2018). VLPs have an enormous potential for use as extremely effective antiviral vaccines, since they mimic the conformation of a viral particle, and are composed of some or all of the structural proteins of the virus, but without the viral genome. Therefore, they form a non-replicating particle, preventing the possibility of reversion and pathogenic infection, which makes them safe as vaccines. In addition, they provide a high cellular and humoral immune response, as they stimulate the immune system through the recognition of repetitive subunits of the antigen in question (Roldão et al., 2010). Several VLP-based vaccines are now commercially available, but none of them are against arboviruses. This study describes a methodology to produce VLPs from the ZIKV containing its C, prM and E structural proteins produced in insect cells using the baculovirus-derived expression system. To this end, we chose the commercial Bac-to-Bac system because of its wide use and facility and speed in generating recombinant products (Liu et al., 2013). Standardization of the use of recombinant baculovirus produced in insect cells as an expression system to produce VLPs represents a promising strategy to develop a platform for producing vaccines against arboviruses.

The infection assays for the expression of the proteins of interest were conducted with the BV- CprME -ZIKV obtained from infected Sf9 cells, one of the cell lineages most used in the baculovirus-derived expression system (Wilde et al., 2014). To analyze the expression of the ZIKV envelope protein, we performed immunofluorescence and Western blot analyses. The immunofluorescence experiment showed presence of the E-ZIKV protein in the membranes of the infected cells, enabling characterization of the protein in the cell itself, and thus comparison of protein expression under the MOI of 2 used in the experiment with the negative control, which were Sf9 cells not infected with the BV- CprME -ZIKV. Onset of protein expression is expected to occur after 24 h, since baculovirus polyhedrin is considered a late promoter. In the Western blot experiment, it was possible to observe a band of 55 kDa that corresponds to the size of ZIKV-E protein. In the positive control sample, IN-ZIKV, it was possible to observe a band slightly above 55 kDa. This can be explained because the inactive parental virus used as a positive control was produced in mammalian cells, which would lead to a different glycosylation pattern of the protein compared with that of the protein produced in insect cells (Zitzmann et al., 2017). The same pattern has been observed in other studies (Wagner et al., 2014), without harming the generation of VLPs.

Our study also standardized the infection for VLP production with MOI 2, unlike the other articles that use MOI5 (Dai et al., 2018; Luo et al., 2020). The value we use represents a 40% reduction in the amount of virus required to produce VLPs. This parameter is very critical in the scaling phases for commercial production and can economically impede the viability of production by this platform. Our process can further be improved and further reduce the viral load required for infection and VLP production, allowing for scaling up.

After the infection assay for the production of ZIKV-VLPs, we proceeded with the steps of concentration and purification. One of the challenges of using the baculovirus expression system is precisely the fact that we produce baculoviruses together with VLPs in this process. Thus, it is important to choose a purification method that is efficient in properly separating these particles. For this, we tested two purification methods by gradient and ultracentrifugation: the sucrose gradient and iodixanol gradient (OptiPrep™). Both are conventional methods used for this purpose as described in the literature (Strobel et al., 2015; Liu et al., 2016). In the sucrose gradient, it was possible to observe the formation of a halo in the gradient close to fraction F6, where the VLPs would be concentrated. In Dot blot assays using the MAb 4G2, it was possible to observe a more intense staining in fraction F6, confirming the concentration of VLPs in the halo formed. The Western blot assay was performed with fraction F6 and marked with the MAb 4G2, evidencing the E-ZIKV protein in the corresponding size of ∼55 kDa. In Dot blot assay using the MAb gp64, it was possible to observe a more intense staining in fractions F6 and F7, evidencing a higher concentration of baculoviruses in the same fraction where the VLPs were also concentrated. This shows that we were unable to achieve total purification with the sucrose gradient, since both VLPs and baculoviruses were concentrated in the same fractions. Dai et al. (2018) produced VLPs from the ZIKV using the baculovirus expression system, but also detected VLPs and baculoviruses concentrated in the same fractions after the sucrose gradient purification process; however, this did not affect the immune response generated by the VLPs produced in immunogenicity tests performed on *in vivo* experiments, which demonstrates that, although the sucrose gradient was not efficient to fully separate the VLPs produced from the baculovirus E-ZIKV, it would still be possible to continue the antigenicity and immunogenicity analyses of the generated particles.

The other method of concentration and purification of VLPs chosen for this study was the iodixanol gradient using the commercial reagent OptiPrep™. The iodixanol gradient presents better rates of purification and yield compared with those of other methods, such as sucrose and cesium chloride (CsCl) gradient (Strobel et al., 2015). It was possible to observe a halo in fraction F7 of the gradient where the VLPs would be more concentrated. Results of the Dot blot analysis using MAb 4G2 showed an intense staining in fraction F7 and almost no marking in the other fractions. Through the Western blot analysis, it was possible to confirm the presence of the E-ZIKV protein by the band formed at the expected size of 55 kDa in fraction F7. Through the Dot blot analysis using MAb gp64, it was possible to observe a more intense staining in fraction F6, evidencing a higher concentration of baculoviruses in that fraction. However, it was possible to observe staining also in fraction F7, where the VLPs were also more concentrated, demonstrating that the iodixanol gradient was also not efficient to fully separate the VLPs produced from the BVs- CprME -ZIKV.

The VLPs concentrated by both the sucrose and iodixanol gradients were characterized by TEM. It was possible to observe VLPs in sizes between 50 and 65 nm in both gradients, and they resemble the parental ZIKV in morphology, size, and surface, as well as the presence of baculoviruses in sizes between 150 and 300 nm, but in smaller number in the iodixanol gradient compared with the sucrose gradient, showing that the purification technique using the iodixanol gradient was more effective in separating the particles and concentrating the VLPs. The IEM assay using antibodies conjugated with gold beads was performed to further characterize the surface composition of the VLPs, and it was possible to observe gold particles surrounding the VLPs, indicating the exposure of the E-ZIKV protein on the external surface of the VLPs purified by the two gradients used. Protein E of flaviviruses is considered the immunodominant epitope among flaviviruses because of its ability to produce neutralizing antibodies (Lindenbach and Rice, 2001). Therefore, we were able to prove the antigenicity of the particles produced and the possibility of inducing an immune response that will be tested in future works. An ultrastructural cytochemistry assay was also performed, where it was possible to observe the VLPs generated in the cytoplasm of Sf9 cells infected with baculovirus CprME -ZIKV and inside intracellular vesicles. It was also possible to observe VLPs outside the plasma membrane of the cells, possibly after the budding process, which is similar to the exocytosis process of the parental ZIKV. In the Sf9 cells not infected with baculovirus E-ZIKV used as a negative control, these particles were not observed, but only empty intracellular vesicles, showing that the baculovirus E-ZIKV was able to infect the Sf9 cells, resulting in the production of VLPs that were properly secreted into the extracellular medium.

The results obtained in this work demonstrate the effectiveness in the production and concentration of VLP compounds of E-ZIKV proteins through Sf9 cells infected with the recombinant baculovirus E-ZIKV and further purified using the sucrose or iodixanol gradient method. Although the methods used to purify the VLPs were not able to eliminate all the baculoviruses present in the samples, they can still be used as a polishing step in processes where a certain number of baculoviruses can be tolerated. In this work, we used superficial methods that should be further explored, such as column chromatography and affinity. Some vaccine leaflets, such as the Flublok^®^ Quadrivalent (Protein Sciences Corporation), describes that each 0.5 mL dose may also contain residual amounts of baculovirus and *S. frugiperda* cell proteins (≤19 mcg), baculovirus and cellular DNA (≤10 ng), and Triton X-100 (≤100 mcg). In the description of HPV vaccine Cervarix^®^ (Glaxo Smith Kline) there is no indication of residual recombinant baculovirus. Alternatively, it is possible to inactivate baculovirus through gamma radiation, beta propiolactone or formaldehyde, for example.

The development of a vaccine platform that is considered safe, especially for those who are at high risk of suffering from the effects of ZIKV infection, such as immunocompromised individuals and pregnant women, is of extreme importance and a public health priority. We expect that the results obtained in this study may constitute an important biotechnological tool to develop vaccine methods for the ZIKV and can contribute to the development of new strategies for obtaining other viral vaccines.

## Data Availability

The original contributions presented in the study are included in the article/supplementary materials, further inquiries can be directed to the corresponding author.
